# Use of Oligomeric Formulas in Malabsorption: A Delphi Study and Consensus

**DOI:** 10.3390/nu17091426

**Published:** 2025-04-24

**Authors:** Carmelo Diéguez Castillo, Maryam Sidahi Serrano, Andrea Martín Aguilar, Daniel De Luis Román

**Affiliations:** 1 Specialist in Gastroenterology, Hospital Universitario Torrecárdenas, 04009 Almería, Spain; carmelo89dc@gmail.com; 2 Internal Medicine Department, Infanta Elena Hospital, 21007 Huelva, Spain; 3 Medical Department, Abbott Laboratories, 28050 Madrid, Spain; andrea.martin@abbott.com; 4 Center of Investigation Endocrinology and Nutrition, Valladolid University Clinical Hospital, Medicine School University, 47002 Valladolid, Spain; dadluis@yahoo.es

**Keywords:** oligomeric enteral formulas, malabsorption, Delphi consensus protocols, gastrointestinal symptoms, gastrointestinal tolerance, nutritional management

## Abstract

**Background**: Malabsorption syndrome is characterized by chronic diarrhea, abdominal distension, and malnutrition, thereby complicating its diagnosis and treatment. Oligomeric enteral formulas, designed to facilitate absorption in patients with compromised bowel function, have shown clinical efficacy, though their implementation lacks standardization due to the lack of uniform protocols. **Objective**: To establish a multidisciplinary consensus on the use of oligomeric formulas in patients with malabsorption using a Delphi methodology. **Material and Method**: A Delphi study was conducted with 156 specialists in endocrinology, gastroenterology, oncology, and internal medicine. Two rounds of structured surveys assessed clinical practices, associated symptoms, and the use of oligomeric enteral formulas. Data were analyzed using descriptive statistics and non-parametric tests, defining consensus with a median of ≥7 and an interquartile range of ≤3. Likewise, a Median (MED) score of ≤3 was considered as a consensus to reject the statement, while an Interquartile range (IQR) of ≥4 or a MED of 4–6 was considered as no agreement. These statements were reviewed and included in the second round. **Results**: Screening for malnutrition is widely supported (79%), but only 38% of participants reported having specific management protocols. Symptoms such as abdominal distension, abdominal pain, and diarrhea were identified as key predictors of intolerance to polymeric formulas, establishing oligomeric enteral formulas as first choice in these cases. In addition, the effectiveness of an approach that progresses from oligomeric to polymeric enteral formulas once symptoms have stabilized was highlighted. The need for standardized protocols was recognized as a priority to guide nutritional assessment and treatment in patients with malabsorption. **Conclusions**: This consensus reinforces the importance of implementing specific clinical protocols for the nutritional management of malabsorption, including the initial use of oligomeric enteral formulas in patients with severe symptoms and their controlled transition to polymeric enteral formulas.

## 1. Introduction

Malabsorption syndrome is a broad condition that can be caused by various health problems such as inflammatory bowel diseases like Crohn’s disease or ulcerative colitis, surgical resections, or specific metabolic conditions [1,2,3,4]. In particular, the prevalence of malnutrition in inflammatory bowel disease (IBD) patients ranges from 6.1% to 69.7%, depending on various factors [5]. In newly diagnosed ulcerative colitis (UC) patients, overall malnutrition prevalence was 32.4% [6]. Malabsorption is a common complication following various types of surgical resections, particularly in gastrointestinal, colorectal, and bariatric surgeries. After oesophago-gastric cancer surgery, malabsorption is prevalent, affecting 78% of patients post-gastrectomy and correlating strongly with a reduced quality of life [7]. In esophageal and gastric cancer survivors, malabsorption is evident in 73% of patients, with 44% showing pancreatic insufficiency [8]. Bariatric surgeries, especially malabsorptive procedures like the Roux-en-Y gastric bypass (RYGB), can lead to significant nutritional deficiencies [9]. Despite its clinical relevance, there is no clear consensus on the symptoms that define it, as these can vary considerably according to etiology and severity.

Manifestations such as chronic diarrhea, unintentional body weight loss, specific nutritional deficiencies, and steatorrhea are frequently associated with malabsorption but are not exclusive to this condition, making it difficult to diagnose and treat uniformly [4].

Malabsorption and maldigestion represent significant challenges in managing patients with gastrointestinal diseases, gastrointestinal surgeries, and serious or critical illnesses, greatly increasing the risk of malnutrition [10]. Nutritional management plays a crucial role in ensuring recovery and improving patient quality of life. Among the available strategies, oligomeric medium chain triglycerides (MCT) and peptide-based nutritional formulas have proven to be effective alternatives, facilitating digestion and absorption in cases of compromised intestinal function [11,12].

The European Society for Clinical Nutrition and Metabolism (ESPEN) guideline on clinical nutrition in acute and chronic pancreatitis recommends that semi-elemental formulas with medium-chain triglycerides can be used if standard formulas are not tolerated [13]. Recent studies suggest that peptide-based enteral formulas not only optimize gastrointestinal tolerance but also contribute to a significant improvement in clinical outcomes, including a reduction in the incidence of gastrointestinal complications and on days with adverse effects in intensive care settings [8]. Oligomeric enteral formulas are particularly relevant in critically ill patients, where their composition allows for an efficient and safe nutritional supply [4,13,14,15,16].

The structure and function of these formulas are based on proteins that have been previously hydrolyzed into di- and tri-peptides, which facilitates their absorption by specific transporters such as PepT1. Additionally, medium-chain triglycerides (MCTs), as they do not require emulsification or lipolysis, are directly absorbed and provide a rapidly usable energy source. This design not only improves the bioavailability of essential nutrients but also reduces the gastrointestinal adverse effects common in other enteral nutrition approaches [17].

Despite the documented benefits, challenges related to the adoption of these oligomeric formulas persist, particularly regarding economic costs and cost-effectiveness. In addition, while the available evidence is promising, further studies are required to support widespread use in clinical practice [18,19].

This study seeks to develop a multidisciplinary consensus establishing recommendations for the use of oligomeric enteral formulas in malabsorption, considering not only clinical efficacy and safety but also economic viability. The objective is to optimize the nutritional management of patients with complex needs, promoting an evidence-based approach that can be implemented in various health settings.

## 2. Material and Method

This study was conducted using the Delphi method. A prospective consensus study was developed using a Delphi process.

Delphi is an effective structured process to assess cost, effectiveness, applicability, and sustainability in a medical setting and is widely used in obtaining results in clinical settings in which there is great uncertainty. The Delphi approach aims to achieve consensus through the collection of stakeholder opinions and is particularly useful in clinical situations where information or consensus is lacking [20].

### 2.1. Phase 1

A coordinating group was set up to inform the development of the various stages of this study and to discuss the results at each phase. The coordinating group, consisting of endocrinologists, gastroenterologists, and internal medicine physicians with a strong training in clinical nutrition, proposed, defined, and justified the existing controversies on certain aspects related to malabsorption syndrome, the symptoms that form it and that impact malnutrition in these patients, as well as the use of nutritional supplements (oligomeric and polymeric enteral formulas) in intestinal diseases when they are associated with malabsorption.

To prepare a preliminary list of assessments for a Delphi survey, a literature review was conducted on malabsorption syndrome, associated symptoms, and nutritional management of malabsorption syndrome in clinical practice.

### 2.2. Phase 2

The coordinating group prepared a document that included descriptive questions about clinical practice in these disorders and 23 questions encompassing 49 items, focusing on aspects such as the existence of clinical protocols for nutritional management of malabsorption, which symptoms are the most characteristic (vomiting, abdominal distension, nausea, increased gastric residues, abdominal pain, diarrhea, and reflux), which factors influence nutritional status, when to start treatment with an oral nutritional supplement, and in which cases to treat with oligomeric formulas in relation to the conditions with most frequent malabsorption.

The clinical conditions included in this study related to malabsorption syndromes were intestinal resection (short bowel syndrome, bariatric surgery), mechanical intestinal obstruction (Crohn’s stenosis, extensive adhesions, or peritoneal carcinomas), intestinal fistulas (Crohn’s disease, diverticular disease, pancreatic disease, radiation enteritis, colon cancer, ovarian cancer, small bowel cancer), intestinal dysmotility (diabetes, ileus, systemic scleroderma, amyloidosis), small bowel mucosal disease (Crohn’s disease, Celiac disease, radiation enteritis, autoimmune enteropathy, ulcerative colitis, amyloidosis, giardiasis, Whipple disease), exocrine pancreatic insufficiency (unresectable pancreatic cancer, chronic pancreatitis, cystic fibrosis, treatment with somatostatin analogues) and vascular disease (mesenteric vascular failure and mesenteric ischemia) [4].

### 2.3. Phase 3

This study included 156 physicians from various specialties (Table 1), all with extensive experience—approximately 12 years—in managing digestive conditions characterized by malabsorption. Considering that, in our clinical practice setting (Spain), these disorders are predominantly managed within clinical and hospital nutrition services—where endocrinologists represent a significant proportion of the medical staff—this specialty was more prominently represented in the recruitment for the present study.

Each participant engaged voluntarily in this study, and their answers were coded to maintain their anonymity and confidentiality. A document explaining the project and the survey was sent out to the participants. This Delphi survey consisted of two consecutive rounds.

In the first round, panelists were sent a document explaining the purpose of this study, the Delphi method, and how to complete the survey. All participants were asked to score each question. The first round remained open for 12 weeks, with a 4-week reminder sent to those specialists who had partially or totally completed the questionnaire or who did not complete it. At the end of the first round, all participants completed the survey, and their responses were coded and anonymized in a database.

### 2.4. Phase 4

In round 2, the results of round 1 were analyzed. After the analysis, the coordinating group reviewed the results and assessed the items on which consensus was reached. Assessments without consensus were reviewed and rephrased in a dichotomous manner (Yes/No). Round 2 was open for 4 weeks with weekly reminders for participants to complete this final phase. At the end of this last round, all participants completed the survey, and their responses were coded and anonymized in a database. With the results of both rounds, these results were found.

### 2.5. Scoring Method

In each round, panelists were asked to indicate their level of agreement with each statement using a nine-point scale (1: “strongly disagree” to 9: “strongly agree”). Scores of 1 to 3 (rank 1) were considered as having a low degree of agreement, implantation, or knowledge according to the question; scores of 4 to 6 (rank 2) were considered doubtful; and scores of 7 to 9 (rank 3) were considered as having a high degree of agreement.

### 2.6. Methods of Analysis

For each round, descriptive statistics were used to summarize the results for each question, including the percentage of participants scoring from 1 to 9.

Each statement that reached an agreement consensus was defined as a median consensus score (MED) of ≥7 and as an interquartile range (IQR) of ≤3.

Likewise, a MED score of ≤3 was considered as a consensus to reject the statement, while an IQR of ≥4 or a MED of 4–6 was considered as no agreement.

These statements were reviewed and included in the second round with only two possible responses (Yes/No) and were considered general consensus when 50% or more of the responses were “Yes” or “No”.

### 2.7. Data Analysis

Statistical analysis was performed using the Statistical Analysis System (SAS) software version 9.4. The MED, IQR, and standard deviation were calculated for each statement. A comparison of variables was performed using the non-parametric U Mann–Whitney test, where a *p*-value < 0.05 was considered significant.

## 3. Results

A total of 156 specialists, 62% women and 38% male (62.18%), with a mean age of 41.74 (SD 8.5) years and 12.3 (SD 8.4) years of experience, participated in the project. The most represented specialties were endocrinology (46.51%), followed by oncology (19.87%), and gastroenterology (14.1%) (Table 1).

**Figure 1:**
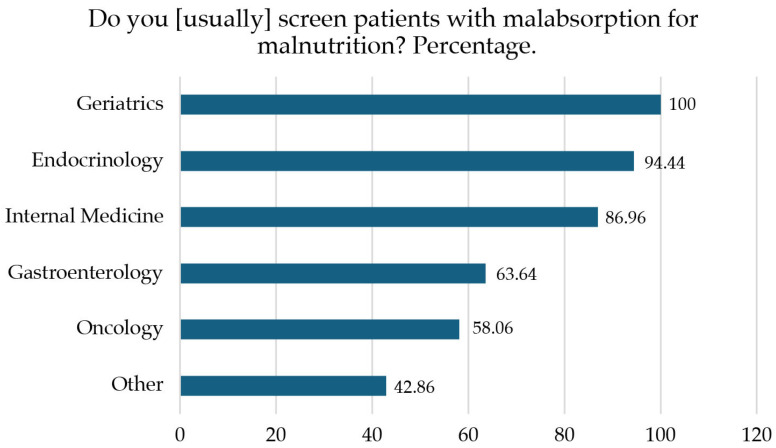
Screening for malnutrition by specialty.

A total of 81% of participants reported that their hospital has a clinical nutrition unit, with no differences between the specialties consulted (*p* = ns). A total of 79% of specialists regularly performed screening for malnutrition in their patients with malabsorption, with differences usually depending on the specialty. Endocrinologists were the specialists who performed the most screens (94.44%), while oncologists performed the least (58.06%) (*p* < 0.0001), Figure 1.

The availability of a nutrition unit in the hospital did not influence patient screening (81.1% vs. 72.4%; *p* = 0.31). Consensus agreement was reached among all participants on the statement that malnutrition screening should be performed in patients with malabsorption (Median 9, IQR 0) (Table 2).

Table 3 shows the screening tools most used by the participants, with the Malnutrition Universal Screening Tool (MUST) (78.2%) and the Mini Nutritional Assessment Short form (MNA-SF) (32.05%) being the most used. Differences were found in the use of some scales between specialties. For example, MNA-SF is more widely used in internal medicine than by oncologists or endocrinologists (60.87%, 35.48%, and 26.39%, respectively; *p* < 0.0006).

A protocol for nutritional management of these patients was not available to 62% of participants at their department, and this percentage was lower in hospitals with a clinical nutrition unit (37.82% vs. 62.18%; *p* < 0.0001). There is a consensus that a protocol should be available for nutritional management of patients (Median 9, IRQ 1) (Table 2). In the same line, 79% of participants did not have a protocol at the site or department for the treatment of malabsorption syndrome, and this was only present in sites with a nutrition unit (25% vs. 0%; *p* < 0001). This aspect was declared a necessity by the participants (Median 9, IRQ 1) (Table 3) with no differences between specialties (*p* = 0.34). A higher percentage (90%) did not have a protocol for quantifying symptoms associated to malabsorption focused on centers with a clinical nutrition unit (11.81% vs. 3.45%; *p* < 0.308) and there is agreement that it should be available (Median 9, IQR 1) (Table 2) with no differences between specialties (*p* < 0.129).

### 3.1. Gastrointestinal Symptoms That Involve Malabsorption Syndrome and Compromise Nutritional Status

In the opinion of the panelist respondents, abdominal distension (Median 8, IQR 2), increased gastric residues (61.5% agreement in round 2), abdominal pain (Median 8, IQR 3), and diarrhea (Median 9, IQR 1) are the main gastrointestinal symptoms included in the malabsorption syndrome, excluding vomiting, nausea, and reflux (consensus of “no agreement” in all of them) (Table 2). Furthermore, vomiting (Median 8, IQR 2), abdominal distension (71.2% agreement in round 2), nausea (Median 7, IQR 3), increased gastric residues (Median 7, IQR 3), abdominal pain (Median 8, IQR 2), and diarrhea (Median 9, IQR 1) are the symptoms that most extensively affect nutritional status, with reflux being left out (“no agreement” consensus) (Table 2).

### 3.2. Oral Nutritional Supplementation (ONS) as Nutritional Therapy in Malabsorption Syndrome

Severe gastrointestinal symptoms influence the type of supplementation prescribed. A total of 90.38% of respondents agree that they usually prescribe an oligomeric enteral formula if symptoms are severe (Median 8, IQR 2), with no differences between specialties (Table 2).

Regarding this, 71% of those surveyed agreed (score 7 to 9) that, according to their experience, patients with malabsorption syndrome have poorer tolerance to polymeric enteral formulas (Median 7, IQR 2) (Table 2), with gastroenterologists showing a mean agreement score slightly lower than the rest of specialists (6.64 SD 1.29 vs. 7.22 SD 1.39 in all other specialties; *p* = 0.0224).

**Figure 2:**
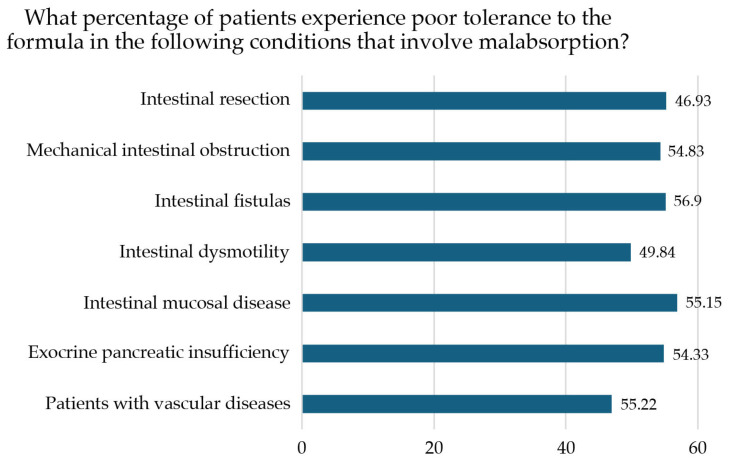
Percentage of patients with poor tolerance of the nutritional formula according to the malabsorption condition.

The proportion of patients with poor tolerance of the polymeric enteral formula in major malabsorption conditions was assessed, with nearly 50% showing poor tolerance across conditions evaluated (Figure 2).

In particular, consensus was reached with a high degree of agreement on the concept that poor tolerance of the polymeric enteral formula often occurs in the case of intestinal resection (62.2% agree, Median 7, IQR 2), mechanical intestinal obstruction (59.6% agree, Median 7, IQR 2), intestinal fistulas (59% agree, Median 7, IQR 2), intestinal dysmotility (53.8% agree, Median 7, IQR 2), intestinal mucosal disease (67.9% agree, Median 7, IQR 2), exocrine pancreatic insufficiency (61.5% agree, Median 7, IQR 3), and vascular disease (53.2% agree in round 2) (Table 2).

It was agreed that the presence and severity of vomiting (56.4% agreement, Median 7, IQR 3), abdominal distension (76.9% agreement, Median 7, IQR 1), nausea (70.5% agreement in round 2), increased gastric residues (58.3% agreement, Median 7, IQR 3), abdominal pain (70.5% agreement, Median 7, IQR 2), and diarrhea (89.1% agreement, Median 8, IQR 1) are predictors of poor tolerance to polymeric nutritional supplementation.

The participants were asked whether the oligomeric enteral formulas were considered as first-line treatment when different gastrointestinal symptoms occurred. The specialists reached a consensus with a high degree of agreement in the case of abdominal distension (74% agreement, Median 7, IQR 2), increased gastric residues (54.4% agreement in round 2), abdominal pain (65.4% agreement, Median 7, IQR 2), and diarrhea (91% agreement, Median 8, IQR 1), while they considered a consensus with “no agreement” in the case of vomiting (45.5% agreement), with differences between the gastroenterologists and oncologists versus the rest (72.7% and 54.8% agreement vs. 45.51% in the rest of specialties; *p* = 0.00380), nausea (42.3% agreement), and reflux (35.3% agreement) (Table 2).

It was agreed that starting nutritional treatment with an oligomeric enteral formula and then switching to a polymeric enteral formula once the patient has recovered from gastrointestinal symptoms (74% agree, Median 8, IQR 2) is a good strategy. In particular, using oligomeric enteral formulas as a first-line treatment and subsequently switching to polymeric formulas is considered an efficient strategy (saving visits and resources) when malabsorption/maldigestion is suspected in the case of intestinal resection (74.4% agree, Median 7.5, IQR 2), mechanical intestinal obstruction (59.6% agree, Median 7, IQR 3), intestinal fistulas (73.1% agree, Median 7, IQR 2), intestinal dysmotility (57.1% agree, Median 7, IQR 3), intestinal mucosal disease (75% agree, Median 8, IQR 1.5), exocrine pancreatic insufficiency (66.1% agree, Median 7, IQR 2), and patients with vascular pathology (50.6%, Median 7, IQR 3) (Table 2).

A total of 75% of those surveyed had started nutritional treatment with an oligomeric enteral formula, followed by conversion to a polymeric enteral formula after the patient’s gastrointestinal symptoms had recovered. Endocrinologists and internal medicine physicians had started treatment in the greatest proportion (86.1% and 82.6%; *p* = 0.0018). Overall, this treatment lasted 12.09 (SD 35.1) weeks. Table 4 shows the reported duration in which the oligomeric enteral formula was prescribed during treatment of the different pathologies tested.

## 4. Discussion

To the best of our knowledge, this is the first study seeking to establish consensus on the role of oligomeric enteral formulas in the nutritional status of patients with malabsorption syndrome, using the Delphi methodology.

There was a unanimous consensus that screening for malnutrition should be routinely performed in patients with malabsorption. However, only 79% of participants reported that they usually did so, with significant differences between specialties, reflecting a gap between ideal practice and clinical reality. Notably, there is a general lack of specific protocols for the nutritional management of patients, particularly for quantifying symptoms and providing nutritional treatment in conditions with malabsorption. This could be one of the reasons for the observed percentage. This issue explains the consensus among all specialties on the need to create a protocol or algorithm for classification and nutritional treatment that also addresses the role of associated symptoms to guide medical care. The difference in the performance of screening among specialties may be due to various structural and organizational circumstances of hospital departments or the training of professionals in those departments. These aspects are difficult to assess with our current study design. Our Nutrition Unit functions as a transversal care unit for the care of admitted patients, carrying out nutritional assessment, adequate nutritional support, and the transition to hospital discharge with subsequent care continuity. However, it is important to note that hospital organization models can vary widely across Spain. In other institutions, Nutrition Units may be integrated within specific departments, may not function transversally, or may have limited roles in patient follow-up after discharge. These differences in structure and clinical pathways may also contribute to variability in screening performance and nutritional management outcomes.

The most used tools are MUST (78.2%) and MNA-SF (32.05%), with variations depending on the specialty [21,22]. Based on the above items, it would be advisable to prioritize malnutrition screening using a scale that uses few easy-to-apply items for an initial assessment without the need for additional tests; this scale should be valid for both inpatients and outpatients to facilitate and compare the course over time. To date, there is no clear recommendation on which screening tool to use for this type of patient. In this context, it would be useful to promote additional research comparing the effectiveness of existing tools, specifically in the population of patients with malabsorption, or to establish a consensus based on the experience with their use [23].

Several symptoms appear to be clearly associated with malabsorption syndrome, such as abdominal distension, increased gastric residues, abdominal pain, and diarrhea. Other symptoms such as vomiting or nausea remain outside the clinical signs of malabsorption perceived by respondents. These findings coincide with the known pathophysiology of malabsorption, where the compromise in digestion and absorption of nutrients frequently translates into these gastrointestinal signs [24].

However, regarding the influence on nutritional status, vomiting and nausea, along with other malabsorption symptoms, are important to consider in managing potential nutritional risk for the patient. This is likely due to their ability to limit oral intake and cause the loss of fluids and electrolytes. Therefore, they are essential components in the comprehensive nutritional assessment and management for personalized nutritional care [25].

Regarding nutritional treatment, we evaluated the percentage of patients with malabsorption who showed intolerance to polymeric enteral formulas in the main associated diseases. The results indicated that approximately 50% of cases presented tolerance difficulties in all clinical conditions involving malnutrition. In the case of intestinal resection, including short bowel syndrome and bariatric surgery, it was recognized that anatomical and functional changes that compromise intestinal absorption predispose a low tolerance of polymeric formulas. Likewise, conditions of mechanical intestinal obstruction, such as Crohn’s disease strictures or postoperative adhesions, were also associated with significant difficulties in processing polymeric formulas due to altered motility and intestinal transit. In patients with intestinal fistulas, particularly those related to chronic inflammatory diseases or surgical complications, nutrient absorption is severely limited, making polymeric enteral formulas less likely to be tolerated. Furthermore, intestinal dysmotility disorders, such as paralytic ileus and disorders associated with systemic diseases such as diabetes mellitus or scleroderma, generate a gastrointestinal environment where digestion and absorption of complete enteral formulas is suboptimal. Intestinal mucosal diseases such as Crohn’s disease, Celiac disease, and autoimmune enteropathies are characterized by inflammation and structural damage of the intestinal surface, making it difficult to process complex components of polymeric enteral formulas. Finally, in exocrine pancreatic insufficiency, a lack of digestive enzymes limits the ability to break down macronutrients, particularly fats and proteins, which aggravates intolerance. In cases such as cystic fibrosis, it is recommended to always include replacement enzyme therapy [26,27].

This consensus emphasizes the need to consider nutritional alternatives such as oligomeric enteral formulas in these clinical scenarios to ensure greater tolerance and optimize nutrient absorption in patients with severe malabsorption [15]. Symptoms such as abdominal distension, diarrhea, and abdominal pain were identified as primary predictors of poor tolerance to polymeric formulas. These results underscore the importance of personalizing nutritional therapy based on the severity and nature of gastrointestinal symptoms.

Oligomeric enteral formulas were shown to be the first-line treatment for patients with malabsorption and severe gastrointestinal symptoms, with 90% of specialists indicating that they prescribe them in these cases. These formulas improve gastrointestinal tolerance, which is crucial under critical conditions. Several studies support this, and there is consensus among the specialists surveyed [14,28].

The use of oligomeric enteral formulas as first-line treatment in patients with suspected malabsorption/maldigestion syndrome was widely supported by the surveyed specialists, particularly in relation to certain gastrointestinal symptoms. A clear consensus was reached for its use in cases of abdominal distension, increased gastric residues, abdominal pain, and diarrhea, reflecting the perceived effectiveness of these formulas in relieving symptoms that directly affect nutrient tolerance and absorption. In contrast, vomiting, nausea, and reflux did not achieve consensus as priority indications for the use of these formulas, which could be related to a lower perceived association between these symptoms and the malabsorption process [16]. It is also acknowledged that oligomeric formulas are not uniform; their composition can vary considerably, particularly regarding peptide profiles and medium-chain triglyceride (MCT) content, among other factors. Therefore, the selection of a specific formula should be individualized based on each patient’s tolerance and clinical needs.

One aspect of the consensus was the strategy of starting nutritional support with oligomeric enteral formulas and transitioning to polymeric enteral formulas once gastrointestinal symptoms stabilize. This approach obtained a high consensus, emphasizing it as an efficient option to minimize medical visits and optimize resources. A controlled transition from oligomeric to polymeric enteral formulas in a patient with suspected intolerance requires a gradual shift accompanied by close clinical, anthropometric, and biochemical monitoring by the prescribing specialist. This process should also include careful assessment of the patient’s tolerance to the new formula in order to ensure both nutritional adequacy and gastrointestinal comfort throughout the transition period.

In specific cases such as active phase Crohn’s disease, the ESPEN recommends, given the lack of superiority of some enteral formulas over others, leaving the choice of one type or another to be based on clinical criteria, considering aspects such as the presence of malabsorption and patient tolerability [29]. In pancreatic cancer, there is a recommendation to add nutritional supplements when the requirements are not met despite enzyme replacement therapy without specifying the type of enteral formula [30,31].

There was some agreement as to the mean duration of treatment that varied by disease, ranging from 10 to 14 weeks. The conditions such as intestinal resection (14.4 SD 34.7 weeks) and intestinal mucosal disease (14.1 SD 35.3 weeks) presented the longest periods, reflecting the need for individualized adaptations according to the severity of the disorder.

The cost–benefit ratio and the potential to reduce gastrointestinal complications and hospital admissions could alleviate concerns about their economical cost. However, a controlled transition strategy from oligomeric to polymeric formulas is necessary for these patients [32].

Improving clinical practice in the management of malabsorption disorders requires, first and foremost, a thorough understanding of the symptoms that characterize malabsorption, enabling early identification and, consequently, timely intervention. Clinicians should also be able to recognize early signs of nutritional deterioration in these patients to prevent further complications. Secondly, it is recommended that oligomeric formulas be considered as a first-line option in nutritional intervention, particularly in patients at high risk of intolerance to standard polymeric formulas. Early identification of any signs or symptoms suggestive of poor tolerance to polymeric formulas is essential in guiding appropriate and individualized nutritional support strategies.

The results of this consensus could serve as a basis for the development of a practical algorithm to identify nutritional risk in patients with suspected malabsorption. However, clinical efficacy and applicability should be addressed in its implementation.

First, identifying the number, type, and severity of symptoms is key. There is not always a direct correlation between the number of symptoms and the probability or severity of the malabsorption condition. For example, a patient with a single significant symptom, such as persistent diarrhea, may be at greater risk of malabsorption than a patient with multiple milder symptoms. This highlights the need to prioritize the quality and clinical relevance of symptoms rather than their absolute number.

The screening tool to be used is another consideration, as there is no clear consensus. Tools such as the MUST and the MNA-SF have been widely used, but their use varies depending on specialties, causing discrepancies in the identification and management of nutritional risk in these patients.

The follow-up interval to be used for the patients should also be considered. The recommended follow-up interval, around six months, also raises doubts. Although this period may be based on previous guidelines or standard clinical practices, there is no universal consensus to support this frequency. Earlier follow-up, for example, at three months, could be useful in high-risk cases or patients with rapid symptom progression. The ideal timing could be adjusted based on the patient’s specific condition, response to initial therapy, and dynamics of symptoms.

The difficulty in grouping diseases would be another factor for creating a single algorithm. Diagnosis and management of malabsorption are complicated by the diversity of conditions that share a common pathophysiological basis. Although these conditions may involve similar changes in nutrient absorption, their clinical manifestations, complications, and therapeutic requirements may vary significantly. Pooling them into a single algorithm may simplify the initial evaluation but may also result in a loss of specificity for certain patient subgroups.

Despite these challenges, the results of this consensus represent a fundamental step towards optimizing the nutritional management of malabsorption. They provide the basis for substantial improvement in nutritional risk management in patients with malabsorption, with the potential to improve clinical outcomes and patient quality of life.

## 5. Conclusions

The consensus highlights the importance of prioritizing malnutrition screening in patients with malabsorption and the need for practical and standardized protocols for its implementation.

Abdominal distension increased gastric residual volume, abdominal pain, and diarrhea were identified as key symptoms for the diagnosis and management of malabsorption syndrome. Although other symptoms such as vomiting and nausea are not considered essential for defining the syndrome, they do become important for assessing nutritional risk because of their impact on intake and the general condition of the patient, allowing for more comprehensive and personalized care.

The efficacy of oligomeric enteral formulas as a first-line treatment in enteral support in patients with malabsorption is emphasized, especially in the presence of severe gastrointestinal symptoms. The controlled transition of oligomeric to polymeric enteral formulas was validated as an efficient and adaptable approach according to the severity and course of the patient in cases suspected of poor tolerance.

In conclusion, the consensus findings represent a key step towards standardizing and improving clinical practice in the management of malabsorption, with the potential to positively impact clinical outcomes and patient quality of life. In the future, an algorithm format may be needed, including recommendations on follow-up frequency, transition to polymeric formulas, and personalized management based on the underlying disease. This would help standardize and enhance clinical care for patients with malabsorption.

## Figures and Tables

**Table 1:** Characteristics of participants in the Delphi study.

Variable		N	%
Sex	Male	59	(37.82)
	Female	97	(62.18)
Medical specialty	Endocrinology	72	(46.15)
	Oncology	31	(19.87)
	Gastroenterology	22	(14.10)
	Internal Medicine	23	(14.74)
	Geriatrics	1	(0.64)
	Other	7	(4.49)
**Variable**		**Mean (SD)**	
Age	Years	41.74 (8.52)	
Experience ^1^	Years	12.43 (8.4)	

^1^ Years of experience in the practice of your specialty (after the period of residency or specialty training).

**Table 2:** Questions asked with the outcome of the different rounds and the outcome of consensus among the participants.

Question Text	Round 1	Round 2	Consensus (Yes/No)
**Do you think that patients with malabsorption should be screened for malnutrition?**	Median 9 (IQR 0)		Yes
**Do you think a protocol should be available in your ** **department for the nutritional management of your ** **patients?**	Median 9 (IQR 1)		Yes
**Do you think there should be some type of protocol for the treatment of malabsorption syndrome?**	Median 9 (IQR 1)		Yes
**Do you think there should be a protocol for quantifying the symptoms associated with malabsorption?**	Median 9 (IQR 1)		Yes
**In your opinion, which of the following symptoms make up malabsorption syndrome?**			
Vomiting	Median 5 (IQR 3)	39.7%	No
Abdominal distension	Median 8 (IQR 2)		Yes
Nausea	Median 6 (IQR 3)	48.1%	No
Increased gastric residues	Median 6.5 (IQR 3)	61.5%	Yes
Abdominal pain	Median 8 (IQR 3)		Yes
Diarrhea	Median 9 (IQR 1)		Yes
Reflux	Median 5 (IQR 3)	27.6%	No
**In your opinion, which of the following symptoms would most affect nutritional status?**			
Vomiting	Median 8 (IQR 2)		Yes
Abdominal distention	Median 6 (IQR 2)	71.2%	Yes
Nausea	Median 7 (IQR 3)		Yes
Increased gastric residues	Median 7 (IQR 3)		Yes
Abdominal pain	Median 8 (IQR 2)		Yes
Diarrhea	Median 9 (IQR 1)		Yes
Reflux	Median 6 (IQR 2)	38.5%	No
**Do you usually modify the type of nutritional ** **supplementation based on the severity of the current ** **gastrointestinal symptoms by prescribing oligomeric ** **formulas if the symptoms are severe?**	Median 8 (IQR 2)		Yes
**In your clinical experience, patients with malabsorption syndrome have poorer tolerance to polymeric formulas.**	Median 7 (IQR 2)		Yes
**In your clinical experience, oligomeric formulas are ** **effective as first choice in enteral support for patients with ** **suspected malabsorption/maldigestion syndrome with:**			
Vomiting	Median 6 (IQR 3)	45.5%	No
Abdominal distention	Median 7 (IQR 2)		Yes
Nausea	Median 6 (IQR 3)	42.3%	No
Increased gastric residues	Median 6 (IQR 3)	54.4%	Yes
Abdominal pain	Median 7 (IQR 2)		Yes
Diarrhea	Median 8 (IQR 1)		Yes
Reflux	Median 5.5 (IQR 3)	35.3%	No
**Do you believe that poor tolerance of the polymeric formula frequently occurs in the following malabsorption conditions?**			
Intestinal resection (short bowel syndrome, bariatric surgery)	Median 7 (IQR 2)		Yes
Mechanical intestinal obstruction (Crohn’s stenosis, extensive adhesions, or peritoneal carcinomas)	Median 7 (IQR 2)		Yes
Intestinal fistulas (Crohn’s disease, diverticular disease, pancreatic disease, radiation enteritis, colon cancer, ovarian cancer, small bowel cancer)	Median 7 (IQR 2)		Yes
Intestinal dysmotility (diabetes, ileus, systemic scleroderma, amyloidosis)	Median 7 (IQR 2)		Yes
Mucosal intestinal disease (Crohn’s disease, Celiac disease, radiation enteritis, autoimmune enteropathy, ulcerative colitis, amyloidosis, giardiasis, Whipple disease)	Median 7 (IQR 2)		Yes
Exocrine pancreatic insufficiency (unresectable pancreatic cancer, chronic pancreatitis, cystic fibrosis, somatostatin analog therapy)	Median 7 (IQR 3)		Yes
Patients with vascular diseases (mesenteric vascular failure, mesenteric ischemia)	Median 6 (IQR 2)	53.2%	Yes
**Do you think the presence and severity of any of the ** **following symptoms are predictors of poor tolerance to ** **polymeric nutritional supplementation?**			
Vomiting	Median 7 (IQR 3)		Yes
Abdominal distention	Median 7 (IQR 1)		Yes
Nausea	Median 6 (IQR 2,5)	70.5%	Yes
Increased gastric residues	Median 7 (IQR 3)		Yes
Abdominal pain	Median 7 (IQR 2)		Yes
Diarrhea	Median 8 (IQR 1)		Yes
Reflux	Median 6 (IQR 2)	39.1%	No
**The use of oligomeric formulas as first choice to ** **subsequently switch to polymeric formulas is an efficient strategy (saving visits and resources) when ** **malabsorption/maldigestion is suspected in case of:**			
Intestinal resection (short bowel syndrome, bariatric surgery)	Median 7.5 (IQR 2)		Yes
Mechanical intestinal obstruction (Crohn’s stenosis, extensive adhesions, or peritoneal carcinomas)	Median 7 (IQR 3)		Yes
Intestinal fistulas (Crohn’s disease, diverticular disease, pancreatic disease, radiation enteritis, colon cancer, ovarian cancer, small bowel cancer)	Median 7(IQR 2)		Yes
Intestinal dysmotility (diabetes, ileus, systemic scleroderma, amyloidosis)	Median 7 (IQR 3)		Yes
Mucosal intestinal disease (Crohn’s disease, Celiac disease, radiation enteritis, autoimmune enteropathy, ulcerative colitis, amyloidosis, giardiasis, Whipple disease)	Median 8 (IQR 1,5)		Yes
Exocrine pancreatic insufficiency (unresectable pancreatic cancer, chronic pancreatitis, cystic fibrosis, somatostatin analog therapy)	Median 7 (IQR 2)		Yes
Patients with vascular diseases (mesenteric vascular failure, mesenteric ischemia)	Median 7 (IQR 3)		Yes
**Do you consider it a good strategy to start nutritional ** **treatment with an oligomeric formula and then switch to a polymeric formula after the patient’s gastrointestinal ** **symptoms are recovered?**	Median 8 (IQR 2)		Yes

**Table 4:** Duration of treatment in weeks with an oligomeric formula according to the malabsorption condition prescribed by respondents.

	N	Mean	SD
Intestinal resection (short bowel syndrome, bariatric surgery)	117	14.42	34.7
Mechanical intestinal obstruction (Crohn’s stenosis, extensive adhesions, or peritoneal carcinomas)	117	13.99	36.3
Intestinal fistulas (Crohn’s disease, diverticular disease, pancreatic disease, radiation enteritis, colon cancer, ovarian cancer, small bowel cancer)	117	10.58	12.1
Intestinal dysmotility (diabetes, ileus, systemic scleroderma, amyloidosis)	117	12.03	35.1
Mucosal intestinal disease (Crohn’s disease, Celiac disease, radiation enteritis, autoimmune enteropathy, ulcerative colitis, amyloidosis, giardiasis, Whipple disease)	117	14.09	35.3
Exocrine pancreatic insufficiency (unresectable pancreatic cancer, chronic pancreatitis, cystic fibrosis, somatostatin analog therapy)	117	14.51	34.8
Patients with vascular diseases (mesenteric vascular failure, mesenteric ischemia)	117	12.09	35.1

**Table 3:** Tools commonly used by the participants to screen for malnutrition.

Scale	N	%
MUST	122	78.21
MNA-SF	50	32.05
NRS-2002	28	17.95
CONUT	15	9.62
GLIM	6	3.85
Other	20	12.82

Malnutrition Universal Screening Tool (MUST), Mini Nutritional Assessment Short form (MNA-SF), Nutritional Risk Screening (NRS-2002), CONUT scale (CONUT), Global Leadership Initiative on Malnutrition (GLIM).

## Data Availability

The data presented in this study are available on request from the corresponding author. The data are not publicly available due to ethical reasons.
